# Optimizing the use of *in vitro* transcribed *SGK1*-mRNA as a therapeutic tool to treat female infertility

**DOI:** 10.1186/s13104-025-07346-5

**Published:** 2025-07-23

**Authors:** Itishri Sahu, Fabienne Engelmann, Brian Weidensee, Harivignesh Ganesan, Sara Y. Brucker, Yogesh Singh, Madhuri S. Salker

**Affiliations:** 1 Department of Pediatrics I–Pediatric Infectiology and Immunology, Translational Genomics and Gene Therapy, University Hospital Tübingen, Wilhelmstraße 27, Tübingen, 72074 Germany; 2 Ziphius NV, Rijvisschestraat 120, 9052 Ghent, Belgium; 3 Department of Women’s Health, University Hospital Tübingen, Calwerstraße 7/6, Tübingen, 72076 Germany; 4 Institute of Medical Genetics and Applied Genomics, University Hospital Tübingen, Calwerstraße 7/6, Tübingen, 72076 Germany

**Keywords:** IVT-mRNA, SGK1, Fertility

## Abstract

**Objectives:**

Gene therapy has emerged as an encouraging therapeutic approach for several diseases. The use of plasmid DNA (pDNA) or viral vectors encoding proteins have yielded low efficacy in recent trials. Synthetic messenger RNA (mRNA), also known as *in vitro* transcribed mRNA (IVT-mRNA), is a highly potent alternative. However, the use of IVT-mRNA in reproductive conditions remains scarce. Our previous work has demonstrated the involvement of endometrial serum- and glucocorticoid kinase 1 (SGK1), where it influences pregnancy outcomes in murine models. On this basis, we investigated the expression and function of human SGK1 encoded by IVT-mRNA *in vitro*.

**Results:**

We show using flow cytometry, quantitative RT-PCR and immunoblotting, that IVT-mRNA has a superior transfection efficiency compared with pDNA. At protein level, we show that the constitutively active SGK1 variant (*SGK1*_S422D_) could increase protein levels and that the inactive SGK1 variant (*SGK1*_K127A_) could reduce SGK1 levels. This effect of SGK1 activity modulation could contribute to reprogramming endometrial cells and support a conducive environment for pregnancy.

**Supplementary Information:**

The online version contains supplementary material available at 10.1186/s13104-025-07346-5.

## Introduction

Reproductive failure remains a significant health challenge, with infertility affecting approximately 10–15% of couples worldwide [1]. Female infertility is a multifaceted issue influenced by a variety of factors, including hormonal imbalances, ovulatory dysfunction, tubal obstructions and uterine abnormalities [2]. Additionally, lifestyle factors such as obesity, smoking and excessive stress can further contribute to infertility. Among these, Advanced Maternal Age (AMA; aged 35 years or older), is one of the most prevalent causes of infertility today. AMA has been linked to decreased fertility due to follicle depeletion and the diminished quality and quantity of oocytes [3, 4]. Moreover, aging is associated with changes in the reproductive system, such as altered hormonal levels, increased oxidative stress and cell senescence, which negatively impacts the correct functioning of the endometrium, thus reducing the chances of conception. Whilst assisted reproductive technologies such as *in vitro* fertilization (IVF) and the use of high-quality donor oocytes might help circumvent issues related to the quality of oocytes in AMA patients, implantation failure remains a common obstacle, indicating that the endometrium may indeed play a pivotal role [5]. Even with viable embryos, successful implantation often hinges on the proper functioning and the correct milieu of growth factors and mediators of the endometrial lining.

Implantation signifies the initial point of physical communication between the maternal endometrium and the developing embryo [6]. However, the molecular mechanisms that govern endometrial receptivity remain poorly understood. Our earlier work has demonstrated the involvement of the Serum and glucocorticoid kinase 1 (SGK1) in the endometrium as a key player in this process, where it influences reproductive and pregnancy outcomes in murine models. The SGK family comprises of three isoforms: SGK1, SGK2, and SGK3. These isoforms share similar biochemical properties and structure, with approximately 80% sequence homology. These kinases also belong to the AGC superfamily [7, 8]. The AGC family includes at least 60 members, which have been extensively studied, such as Protein kinase B (PKB/AKT), Ribosomal protein S6 kinase beta-1(S6K) and Rho-associated coiled-coil containing kinases (ROCK) [9].

SGK1 regulates multiple cellular and physiological processes, including ion transport and cell survival [8, 10–12]. In response to stimulation, mammalian target of rapamycin complex 2 (mTORC2) phosphorylates the hydrophobic motif of SGK1 at serine 422, inducing a conformational change to recruit the master kinase, 3-phosphoinositide-dependent protein kinase-1 (PDK1) [13]. Subsequently, PDK1 phosphorylates the kinase domain of SGK1 at threonine 256 ensuring full activation [13, 14]. Accordingly, activated SGK1 is then able to modulate downstream functions. Substitution of this serine by aspartate (SGK1_S422D_) results in a constitutively active form [13]. Replacement of lysine at position 127 for alanine (SGK1_K127A_) abolishes the catalytic domain leading to enzyme inactivation [15].

We described earlier that endometrial SGK1 levels are higher in women with unexplained infertility [5]. We also verified that an increase of SGK1 activity can abolish implantation and can decrease both the glandular area and epithelial cell width, thus reducing the entire size and weight of the uterus. The menstrual cycle regulates the fluid environment within the uterine lumen which is indispensable for crucial reproductive events, including transport of sperm, embryo development and implantation [5, 16]. Uterine fluid absorption is controlled by epithelial sodium channels (ENaCs) which are found on the apical surface of luminal and glandular epithelial cells, which are regulated by the steroid hormone estrogen [14, 17]. Thus, a continuous increase in SGK1 expression and activity, could in turn could result in the activation of ENaC or other ion channels, potentially causing untimely uterine ‘closure', characterised by apposition of the epithelium to the opposing uterine walls- leading to implantation failure [16].

Despite successfully identifying the role of SGK1 in female infertility, therapeutic manipulation for human applications presents several challenges and potential side effects. One approach that has been explored is the targeted transfection of SGK1 using the Hemagglutinating Virus of Japan (HVJ) [18] envelope vector, as demonstrated by us previously [5]. While HVJ-mediated transduction showed promise in influencing endometrial function, its use is limited by immunogenicity concerns and the potential for insertional mutagenesis. These limitations underscore the need for a more efficient and safer methods of delivering genetic material to the endometrium.

The COVID-19 pandemic has demanded the manufacture of vaccines at record speed. *In vitro* transcribed (IVT) mRNA technology has revolutionized gene delivery systems, offering a more precise and less invasive approach compared with viral vectors. IVT mRNA allows for transient expression of therapeutic proteins without the risks associated with viral integration into the host genome [19]. mRNA-based vaccines have been developed as alternatives to standard vaccine methods [20], as it is rapidly synthesized, standardized and scaled up [20]. This technology has shown great promise in numerous fields, including cancer immunotherapy and vaccine development [19]. By the use of less immunogenic modification, attempts are being made in the field for protein replacement therapies and gene therapies by numerous groups [21]. However, its role in therapeutic use in reproductive medicine remains rudimentary.

In this study, we provide pilot data showing that IVT mRNA technology can effectively deliver *SGK1* to the endometrium, offering a novel therapeutic approach to enhance implantation success in cases of recurrent reproductive failure. By leveraging this advanced technology, we aim to circumvent the challenges posed by viral vectors and provide a safer, more effective means of modulating endometrial receptivity.

## Materials and methods

### IVT mRNA synthesis

SGK1 plasmids were commercially purchased from MRC PPU Dundee, UK and have been previously characterised for their function[5]. SGK1 and its variants were subcloned into the pVAX.A120 vector containing, a poly(A)−120 tail using sticky-end ligation. For control experiments, mKate reporter protein was similarly subcloned into pVAX.A120 vectors. After the cloning procedure was completed, Sanger sequencing was performed to confirm plasmid sequence validation and mutational analysis. The Sanger sequencing files can be found at 10.5281/zenodo.15267264 or with accession number PRJEB88920 (www.ebi.ac.uk). Plasmids were linearized with Xhol (New England Biolabs) downstream of the poly(A) tail prior to *in vitro* transcription (IVT). The IVT reaction was performed using the HiScribe T7 High Yield RNA Synthesis Kit (New England Biolabs Ambion) with co-transcriptional capping using an anti-reverse CAP analog (ARCA, Jena Bioscience) at the 5'end. Purification of the synthesized mRNA was performed with a Monarch Spin RNA Cleanup Kit (New England Biolabs) and was analysed for size and concentration using the Agilent 2100 Bioanalyzer with an RNA 6000 Nano kit (Agilent). For the control a non-template (Ctrl; non-coding) was used.

### Cell culture and transfection

Endometrial Ishikawa cells (#99040201 from Sigma) and HEK293 cells (#CRL-1573 from ATCC) were cultured in DMEM (Sigma) and supplemented with 10% FBS and 1% penicillin/streptomycin. Cells were cultured at 37 °C with 5% CO_2_. To split into plates or to culture, the cells were washed with cold sterile PBS and detached with 5 ml of 0.5% Trypsin–EDTA (Thermo Fisher). Trypsinization was terminated by adding 10% FBS-DMEM. In brief, the cells were transferred to a 50 ml sterile tube and centrifuged at 500 × *g* for 5 min before resuspending in new 10% FBS-DMEM. 24 h before transfection, 250,000 cells/well/1 ml were plated in 12-well plates and left to settle overnight in media without antibiotics. At a confluency of approx. 80%, the media was changed to the reduced serum media, Opti-MEM (Thermo Fisher) and 10% FBS-DMEM at a ratio of 1:4. Following this, the cells were subsequently transfected using 1000 ng IVT-mRNA or equivalent (in nmol) pDNA with Lipofectamine 2000 (Thermo Fisher) following the manufacturer’s instructions. After 5 h, the complexes were removed by replacement with fresh culture medium. Cells were kept for 24 h before downstream analysis. Cells were routinely tested for mycoplasma and for authenticity using STR profiling (ATCC).

### Flow cytometry

The efficiency of transfection of the linear IVT mRNA was measured using flow cytometry. Cells were collected after trypsinisation and subject to flow cytometry. Using a far-red fluorescent marker mKate-2, successful transfection of IVT mRNA and the pDNA was detected using LSR-Fortessa X-20 (BD Bioscience). The success of the transfection was reviewed based on the basis of specific light scattering and fluorescent characteristics, allowing a comparison of transfection efficiency between different methods of transfection (pDNA and IVT-mRNA) compared with a non-transfected control (FlowJo^™^ Software V10.10).

### Quantification of transcribed RNA (RT-q PCR)

Total mRNA was harvested from cells using TRizol^™^ (Invitrogen) and the mRNA concentration was quantified by using a Nanodrop (Thermofisher). 1 µg of RNA was used to synthesize cDNA as per the manufacturers guidelines (Thermo Scientific MaximaTM H Minus cDNA Synthesis Master Mix with dsDNase, Invitrogen) performed on a standard PCR cycler (Bio-Rad). RT-qPCR was performed on the QuantStudio 3 Real-Time PCR System (Invitrogen) by using sets of gene-specific primers and the PowerUp^TM^ SYBR^®^ Green Master Mix (Invitrogen). Primer pairs were purchased from Sigma. The expression levels of *SGK1* were normalised to the housekeeping gene *L19*, ensuring consistent expression across samples. After the amplification run, a melting curve analysis was included to verify primer specificity, confirming the presence of a single amplicon and ruling out our non-specific binding or primer dimers. The relative variations in PCR product were estimated by the ^ΔΔ^CT method [22]. All RT-qPCR experiments were run in duplicates to ensure reproducibility.

**Table:** 

*L19*	Sense (5′-GCG GAA GGG TAC AGC AAT-3′)	Antisense (5′-GCA GCC GGC GCA AA-3′),
*SGK1*	Sense (5′-TTC CTA TCG CAG TGT TTC AGT TCT T-3′)	Antisense (5′-CAC ACT CAC ACG ACG GTT CAC-3′)

### Western blotting

The Western blot procedure for relative protein quantification involved 3 key steps: protein separation by size, electro-transfer, and target protein detection. To begin, the volume required to load 25 µg of protein was quantified using a Bradford assay. Proteins were then separated based on their molecular weight (kDa) using SDS-PAGE with a stacking gel of 4% and 10% separation gel. After electrophoresis, using the wet transfer methods the proteins were transferred on to a PVDF membrane. The membranes were cut according to the protein weight (kDa). Total SGK1 (#07315, 1:1000, EMD Millipore), ENaC (#E4653,1:500, Sigma) and GAPDH (#2118 s, 1:1000, Cell Signalling) were detected using their respective primary and secondary antibodies (anti-Rabbit, 1:2000, #7074s, Cell Signalling) at room temperature for 1 h, followed by washing 3 times with TBS-T. Proteins of interest were detected using a chemiluminescent detection kit (WesternBright™ ECL) and pictured by using iBright™ Imaging System (Invitrogen) at 1–2 min intervals for up to 1 h. The original images were then downloaded from the machine (see supplemental information). Quantification was performed by comparing the relative intensity of each protein band, normalized to the lane's GAPDH signal, using ImageJ [23].

### Statistics and data visualisation

**Fig. 1:**
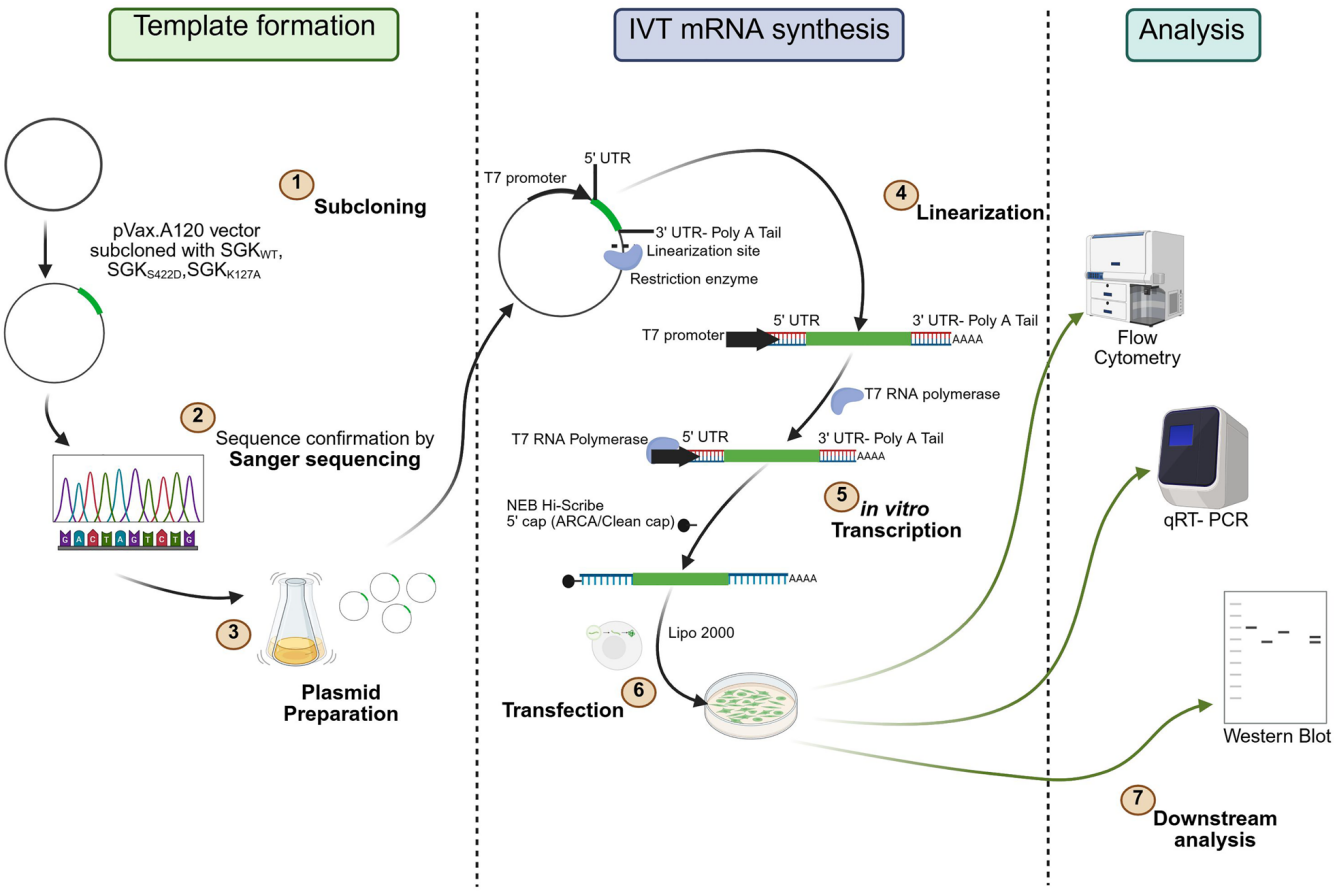
Schematic overview of experimental design. Plasmid with target sequence of interest is transformed (1) is subjected to sequencing (2). Next, the plasmid is amplified and purified (3). Linearization of the plasmid is performed with a restriction enzyme (4), the resulting template DNA is then used for *in vitro* transcription to produce mRNA with co-transcriptional capping (5). The resulting mRNA is transfected with Lipofectamine 2000 (6) and its functionality tested downstream with different methods (i.e flow cytometry, RT-qPCR and Western blotting) (7). Drawn in BioRender

In our analyses, we implemented ordinary one-way analysis of variance (ANOVA) Test with GraphPad Prism (v10.1.1) to test for significance. The data are represented as arithmetic means ± SEM and p ≤ 0.05 was considered statistically significant. N, is the number of biological experiments performed. Figures were made in Inkskape (v1.4) and BioRender (https://BioRender.com/l71x548) for Fig. 1.

## Results

The present study investigated how IVT-SGK1-mRNA can be generated and induced at both mRNA and protein levels. The process of making the IVT-SGK1 mRNA is as follows (Fig. 1): Plasmid containing the sequence of interest is transformed into *E. coli* competent bacteria (1), sequenced for the correct sequence (2), amplified and then purified (3). Next, the workflow goes through sequential steps to achieve the linearized mRNA. The plasmid is linearized with a restriction enzyme (4), the subsequent DNA is then used as a template for *in vitro* transcription producing mRNA (5). Additionally, mRNA needs either co-transcriptional or enzymatic capping, and a poly(A) tail, to be functional. The resulting mRNA is mixed with suitable lipoplexes or nanoparticles, transfected (6), and its functionality tested downstream (7).

**Fig. 2:**
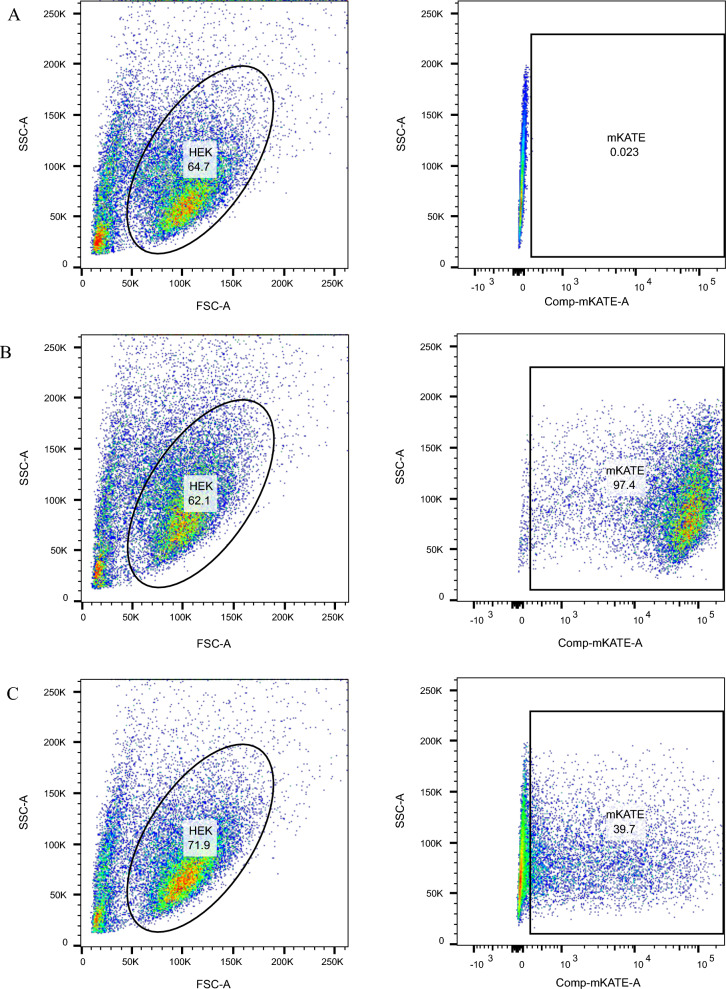
Effective delivery of IVT-*SGK1* mRNA into HEK293 cells. Original FACS plots of (**A**) Gating Strategy. The FACS analysis compared the transfection efficiency of (**B**) IVT-mRNA (97.4%) and (**C**) plasmid DNA (39.7%) to a non-transfected control (n = 3)

We first investigated the expression and function of human *SGK1* encoded by IVT mRNA *in vitro*. We show that IVT-mRNA achieves a transfection efficiency of 97.6%, which is significantly higher than that of pDNA (45.2%), representing a 2.5-fold increase when using HEK293 cells (Fig. 2).

**Fig. 3:**
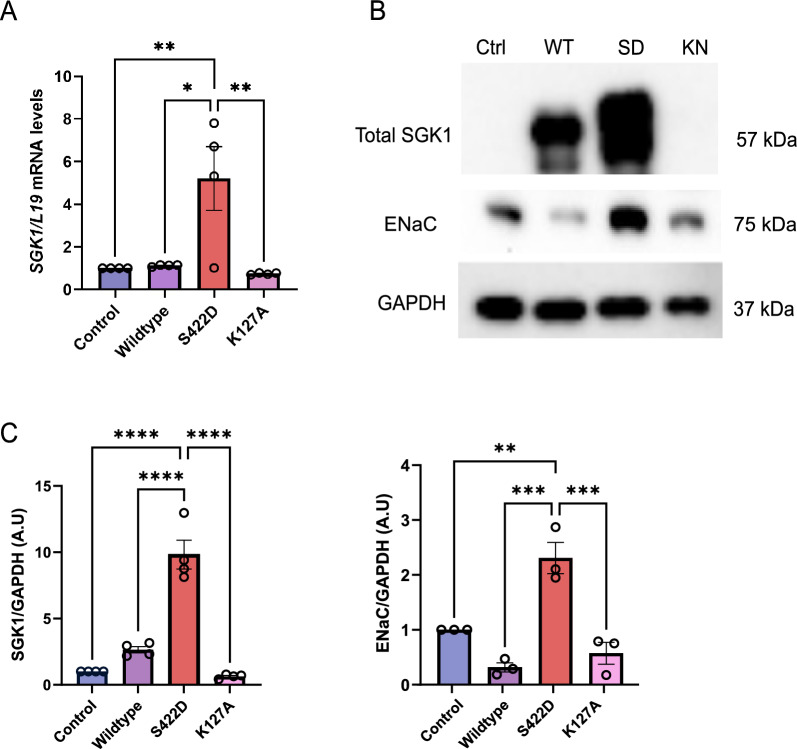
IVT-mRNA induces expression of SGK1 and ENaC. Ishikawa cells transfected with Wild Type (WT), Overexpression (S422D; SD) or inactive (K127A; KN). (**A**) Arithmetic means ± SEM (n = 4) of *SGK1* transcript levels from Ishikawa cells transfected with Wild Type, Overexpression (S422D) or inactive (K127A). The control (Ctrl) was a non-template IVT-mRNA was used and *L19* served as a housekeeping control. (**B**) Original Western blot of SGK1 and ENaC. GAPDH was used as a loading control. (**C**) Arithmetic means ± SEM (n = 3–4) of the respective protein abundance ratios in cell lysate. Significance was determined by using one-way ANOVA* p < 0.05, **p < 0.01 and ***p < 0.001

Since the efficacy of transfection was high, we then used Ishikawa endometrial epithelial cell line as a model [24] to investigate the downstream effect. In the next step, the effect of a 24 h treatment with *SGK1*_WT_, *SGK1*_S422D_, and *SGK1*_K127A_ on the transcript level of *SGK1* in endometrial cells was determined utilizing RT-qPCR. As illustrated in Fig. 3A, treatment of Ishikawa cells with *SGK1*_WT_ resulted in no significant change; *SGK1*_S422D_ led to a five-fold increase in transcript levels, whereas a significant decrease in the transcript levels of *SKG1* was observed following transfection with *SGK1*_K127A_ compared to the wild type and control.

Next, the effect of a 24 h treatment with *SGK1*_WT_, *SGK1*_S422D_, and *SGK1*_K127A_ on the protein expression of SGK1 in endometrial cells was determined utilizing immunoblotting. As illustrated in Fig. 3B and C, treatment of Ishikawa cells with *SGK1*_WT_ resulted in no significant change; *SGK1*_S422D_ led to a four-fold increase in total SGK1 protein levels compared with the wild type and a ten-fold increase compared with the control (non-template), whereas *SGK1*_K127A_ significantly decreased the total SGK1 protein levels.

SGK1 is known to stimulate the Sodium–Potassium ATPase as well as a variety of other carriers and ion channels, including ENaC. The expression of constitutively active SGK1 in the mouse uterus is known to perturb uterine fluid handling and thereby block implantation [5]. Therefore, we tested whether the increase in IVT-*SGK1* mRNA would elevate ENaC protein levels. As illustrated in Fig. 3B&C, ENaC protein levels in Ishikawa cells follow a similar trend to SGK1 protein levels after treatment with *SGK1* IVT mRNA: treatment with *SGK1*_WT_ resulted in no significant change; however, *SGK1*_S422D_ led to a five-fold increase in ENaC protein levels compared with the wild type and a two-fold increase compared with the control, whereas *SGK1*_K127A_ resulted in a significant decrease in ENaC protein levels.

## Discussion

In the late 1980s, the first preliminary investigations of IVT-mRNA revealed that after transfection *in vitro* and *in vivo* mRNA can be immediately translated into a functional form [25]. This was followed swiftly by the application of mRNA in reversal the of diabetes insipidus, albeit it temporarily, unravelling its clinical potential.

The uses of mRNA are highly beneficial due to its exceptional properties; a key point being that mRNA does not require it to be trafficked across the nuclear membrane as it is already active in the cytoplasm [26]. This includes averting the permanent manipulating of the genome, making mRNA a prevalent molecule for protein supplementation therapy [26]. Furthermore, owing to the temporary nature and biodegradability of mRNA, adverse effects can theoretically be avoided [20, 25]. To investigate this potential, we explored whether IVT-*SGK1* mRNA can be synthesized and used to induce or suppress SGK1 expression in the endometrium.

We reported earlier that endometrial SGK1 levels are higher in women with unexplained infertility [5, 17]. The role of SGK1 in regulating early implantation events, pregnancy, and embryo survival makes it a promising clinical target.[14]. We first investigated the expression and function of human *SGK1* encoded by IVT-mRNA *in vitro*. We show that IVT-mRNA has superior transfection efficiency compared to pDNA. An effective delivery system into the cytosol is essential for IVT-mRNA to function as an expression system. In contrast, plasmids need to enter the nucleus, as transcription into mRNA is required. This step makes the transfection process inefficient, resulting in lower transfection efficiency of pDNA than mRNA and poorer efficiency of pDNA to translate *SGK1* [5].

At the protein level, we show that the overexpression (*SGK1*_S422D_) could increase protein levels and *SGK1*_K127A_ could reduce SGK1 levels. The estrogenic phase of the menstrual cycle results in fluid secretion whereas the progesterone dependent phase favours fluid absorption, facilitated by ENaC expressed on the apically facing luminal and glandular epithelial cells [5]. Therefore, overexpression of endometrial SGK1 could stimulate ENaC activity among others. As a consequence, altered uterine fluid composition may lead to a hostile embryonic environment. Furthermore, infertility could be induced by premature uterine ‘closure', characterised by complete apposition of the epithelium of the opposing uterine thereby preventing embryo transport resulting in implantation failure [5, 17]. Furthermore, we show that overexpression (*SGK1*_S422D_) could increase protein levels and *SGK1*_K127A_ could reduce ENaC protein levels.

Therefore, the use of IVT-mRNA could be used to influence the endometrial SGK1/ENaC homeostasis and can be extrapolated for the development of new non-hormonal contraceptives. Furthermore, it is plausible that SGK1 activity in different regions or cell types of the endometrium is regulated by specific phosphatases, which could be inhibited through the development of small IVT-mRNA molecules. This inhibition may render the endometrial layer refractory to embryo implantation. On the contrary, SGK1 inhibitors (heterocyclic indazole derivatives) are currently being trialed for several pathologies, ranging from diabetes and obesity to kidney disorders [27]. Notwithstanding, the development of *SGK1* IVT-mRNA activators or inhibitors could be useful in reproductive medicine in several ways. For instance, prior to embryo transfer, flushing the uterus with an inhibitor could improve the pregnancy rate after IVF. However, the long-term use of such molecules is likely to impair decidual function a consequence that could be harnessed for either a non-hormonal contraceptive or as an alternative strategy for medical termination of pregnancy.

## Limitations

Although our study was conducted *in vitro*, future research should be carried out in murine or larger mammalian models to evaluate its potential for fertility control and to assess any reproductive toxicity concerns. Several caveats of IVT-mRNA need to be addressed including; potential immunogenicity mediated by the immune system (pattern recognition receptors) and nucleases degrading single-stranded mRNA [20, 25]. Solutions such as chemical modifications to reduce immunogenicity, the use of nanocarriers to facilitate cellular uptake and enable tissue- or organ-specific targeting, are rapidly being developed [28].

## Conclusion

In summary, the present study demonstrates proof of concept data that IVT-*SGK1* mRNA is superior in transfection efficacy compared to pDNA. We further demonstrate that the expression of *SGK1*_WT_, *SGK1*_S422D_, and *SGK1*_K127A_ variants differentially modulates protein levels of SGK1 and ENaC, resulting in either increased or decreased expression, respectively. Collectively, these findings highlight a promising non-hormonal approach to selectively facilitate or inhibit embryo implantation. To further validate these results and assess their translational potential, future studies employing *in vivo* models are warranted. Therefore, the testing of IVT-mRNA *in vivo* is required and could create a scalable platform for future therapeutics for unexplained implatation failure.

## Supplementary Information

Supplementary Material 1: Figure S1. Original SGK1 blots uncropped.

Supplementary Material 2: Figure S2. Original ENaC blots uncropped.

## Data Availability

All data is provided within the supplementary information files. Sanger sequencing files can be accessed on 10.5281/zenodo.15267264. the data is also avaliable under study number PRJEB88920 (ERP171977) at the European Molecular Biology Lab -European Bioinformatics institute (www.ebi.ac.uk). Materials can be requested by email to MSS.

## Abbreviations

IVT-mRNA: *In vitro* transcribed-mRNA
SGK1: Serum- and glucocorticoid-regulated kinase 1
ENaC: Epithelial Sodium Channel
