# Supplementary material for: Optimizing the use of in vitro transcribed SGK1-mRNA as a therapeutic tool to treat female infertility
Source: BMC Res Notes. 2025 Jul 23;18:318. doi: 10.1186/s13104-025-07346-5 (PMC12285141; doi:10.1186/s13104-025-07346-5)
Supplement: Supplementary file 2 — Supplementary Material 2: Figure S2. Original ENaC blots uncropped. [file 13104_2025_7346_MOESM2_ESM.pptx]

## Slide 1
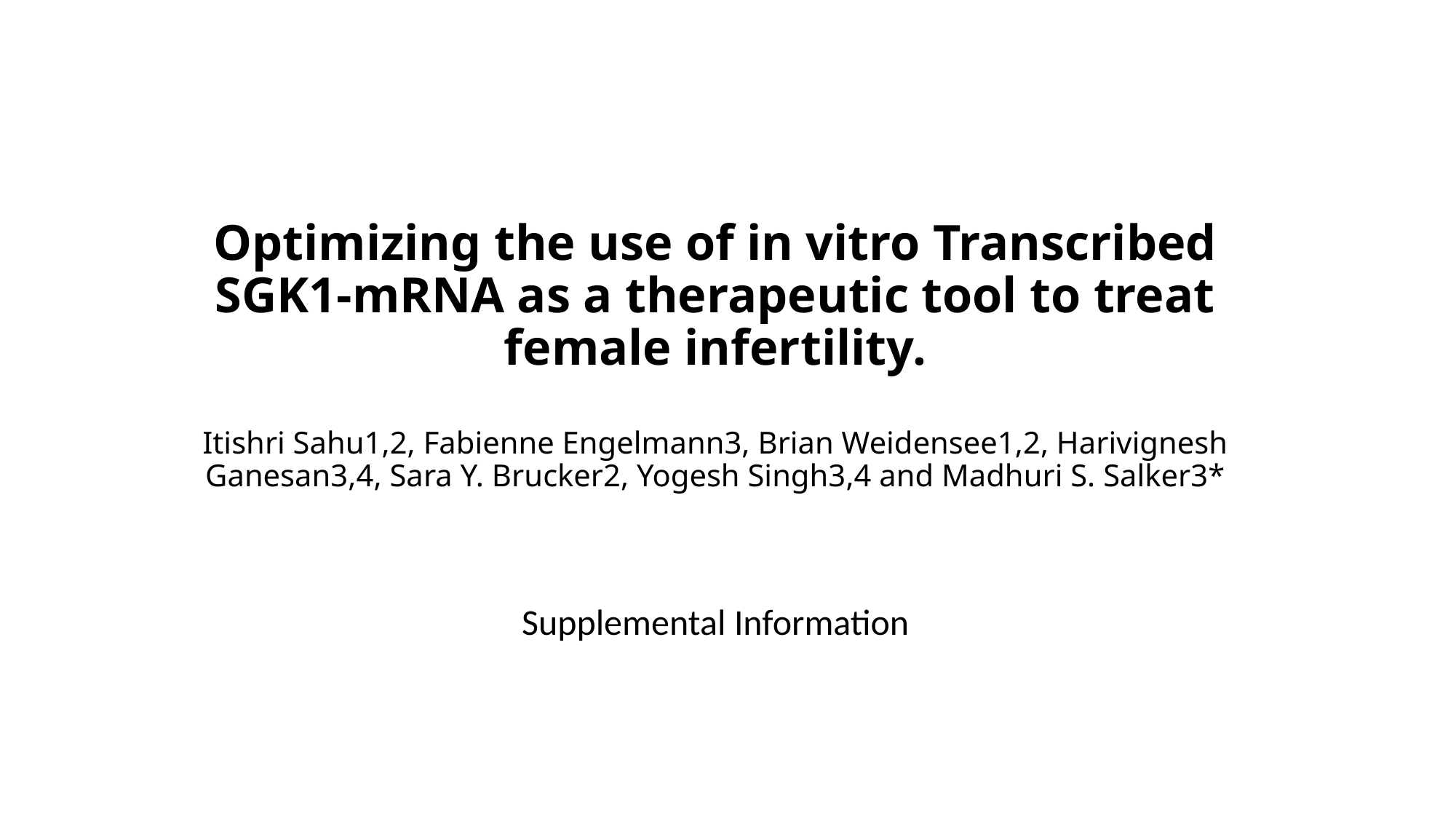

# Optimizing the use of in vitro Transcribed SGK1-mRNA as a therapeutic tool to treat female infertility. Itishri Sahu1,2, Fabienne Engelmann3, Brian Weidensee1,2, Harivignesh Ganesan3,4, Sara Y. Brucker2, Yogesh Singh3,4 and Madhuri S. Salker3*
Supplemental Information

## Slide 2
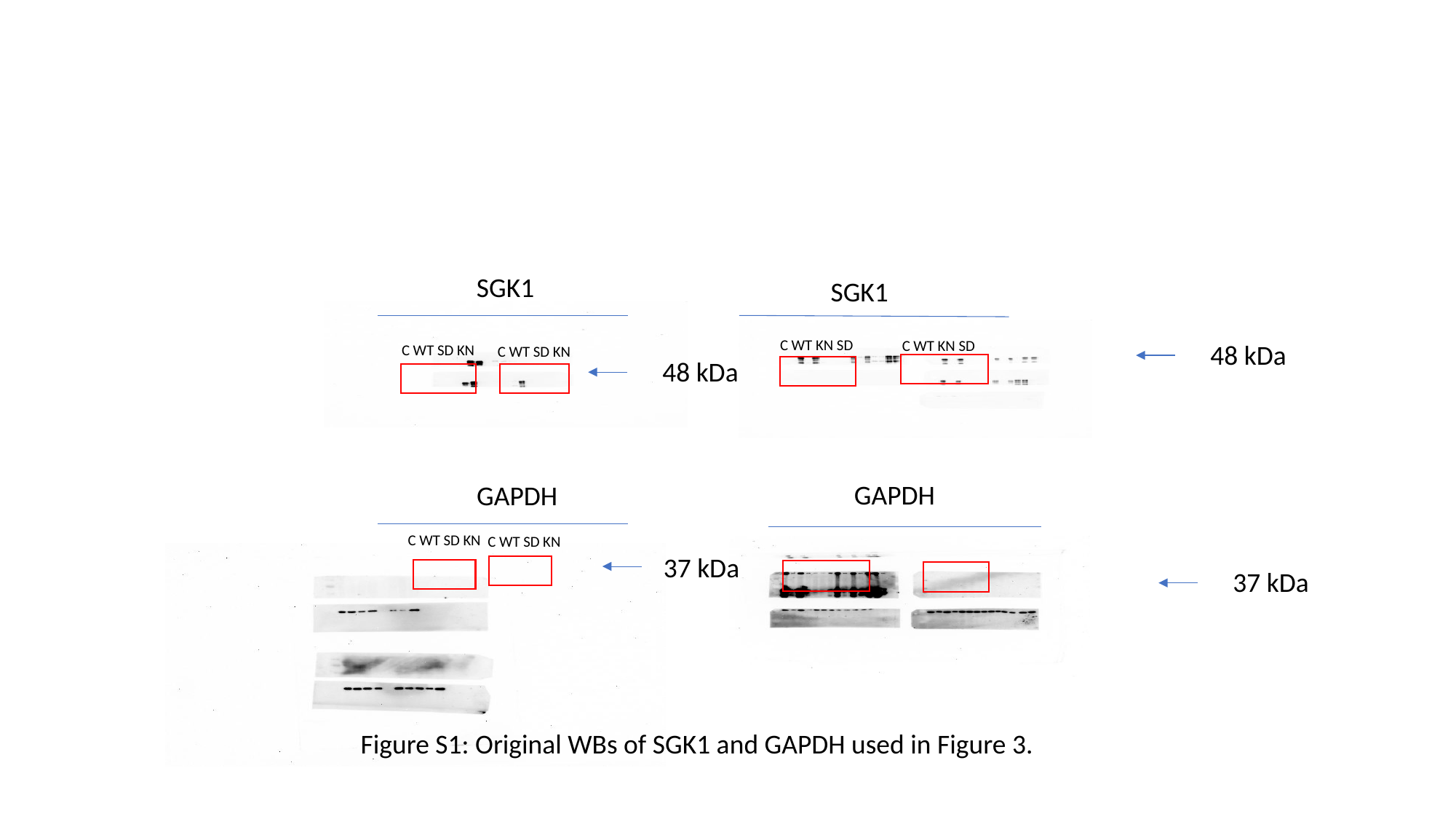

#
SGK1
SGK1
C WT KN SD
C WT KN SD
48 kDa
C WT SD KN
C WT SD KN
48 kDa
GAPDH
GAPDH
C WT SD KN
C WT SD KN
37 kDa
37 kDa
Figure S1: Original WBs of SGK1 and GAPDH used in Figure 3.

## Slide 3
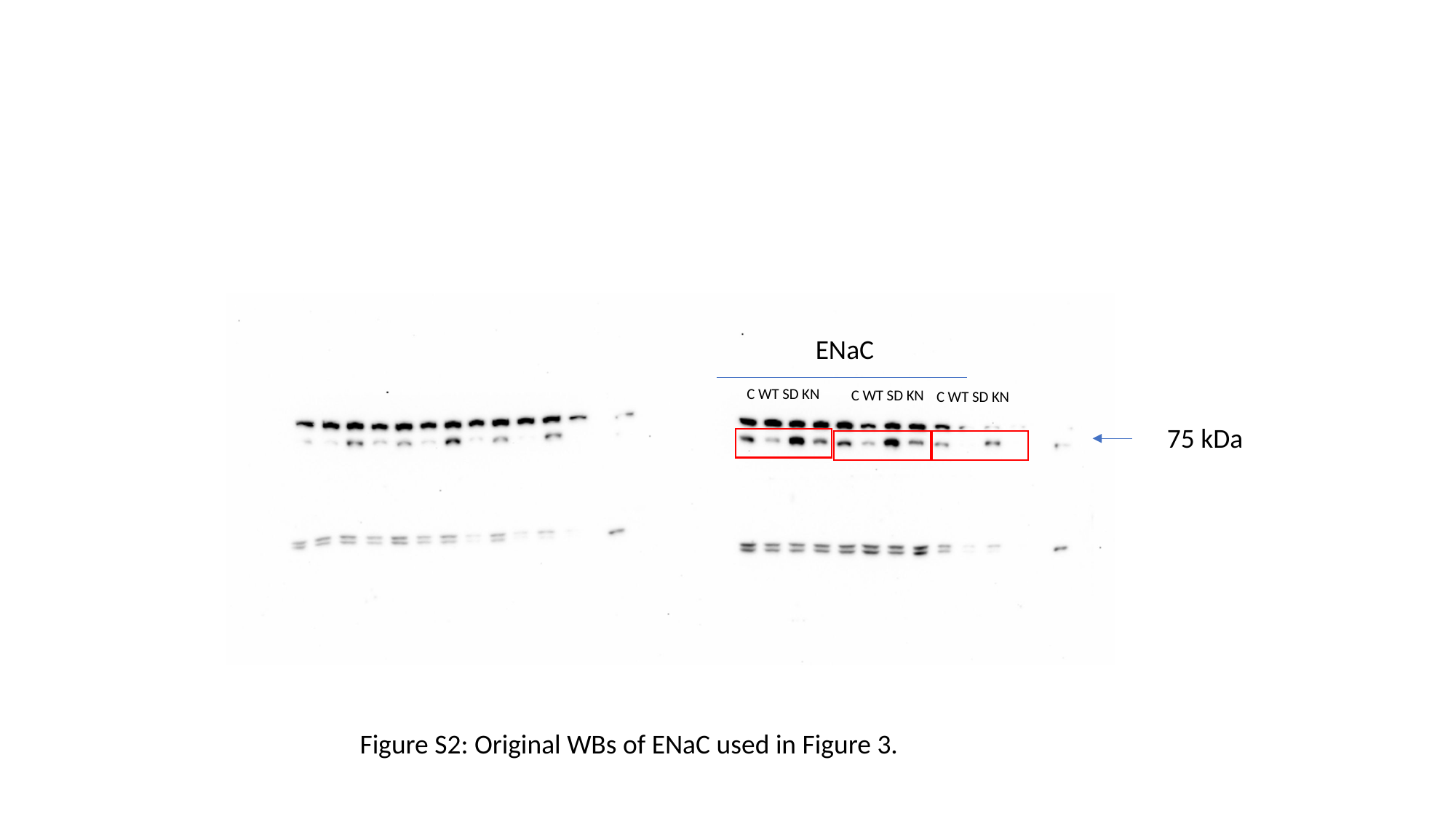

#
ENaC
C WT SD KN
C WT SD KN
C WT SD KN
75 kDa
Figure S2: Original WBs of ENaC used in Figure 3.
